# Theoretical Characterization of the Reduction Potentials of Nucleic Acids in Solution

**DOI:** 10.1021/acs.jctc.0c00728

**Published:** 2021-02-23

**Authors:** Valeria D’Annibale, Alessandro Nicola Nardi, Andrea Amadei, Marco D’Abramo

**Affiliations:** † Department of Chemistry, Sapienza University of Rome, Rome 00185, Italy; ‡ Department of Chemical Sciences and Technology, Tor Vergata University, Rome 00133, Italy

## Abstract

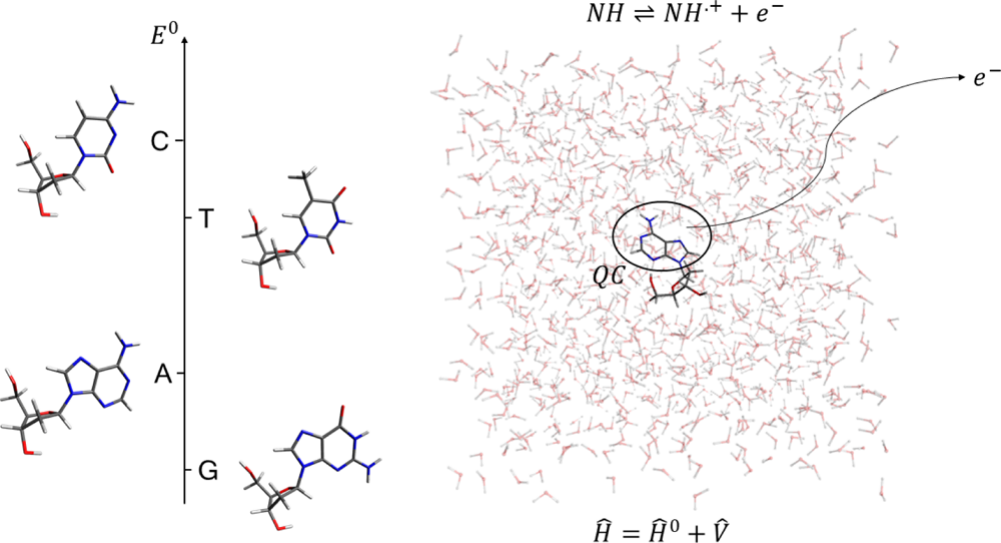

Here, we present the theoretical–computational modeling of the oxidation properties of four DNA nucleosides and nucleotides and a set of dinucleotides in solutions. Our estimates of the vertical ionization energies and reduction potentials, close to the corresponding experimental data, show that an accurate calculation of the molecular electronic properties in solutions requires a proper treatment of the effect of the environment. In particular, we found that the effect of the environment is to stabilize the oxidized state of the nucleobases resulting in a remarkable reduction—up to 6.6 eV—of the energy with respect to the gas phase. Our estimates of the aqueous and gas-phase vertical ionization energies, in good agreement with photoelectron spectroscopy experiments, also show that the effect on the reduction potential of the phosphate group and of the additional nucleotide in dinucleotides is rather limited.

## Introduction

Oxidation of the DNA is involved in very important processes such as DNA damage and long-range DNA charge transfer. The DNA damage induced by the loss of an electron is related to mutagenesis, carcinogenesis, and aging processes,^1−3^ making one of the most effective mechanisms in the loss of information observed in living organisms. On the other hand, the charge transfer along DNA strands has been claimed to explain the cellular mechanisms responsible of the DNA repair^4^ and to design DNA molecules able to function as a molecular wire.^5,6^ Therefore, several studies investigating the effects of the sequence, environment, and structure on the kinetics and thermodynamics of the process have been published. One of the main results of these studies is the relative scale of the nucleobase ionization potentials, where the guanine has shown the lowest ionization potential.^7^ This results in the fact that the guanine is the initial oxidation site of the DNA, that the electron hole migration ends up at a guanine site,^8^ and that the GGG contained in the telomere sequence acts as electron–hole sink.^9^ Concerning the kinetics of the charge transfer, several experimental studies agree with the fact that the charge migration along DNA can occur and that the mechanism can be described as hole hopping between (up to three) stacked bases.^4^ However, the experimental difficulties in the measure of the one electron reduction potential of nucleotides alone and within a DNA strand have partly hindered a complete characterization of such a process. Its determination via electrochemical approaches suffers by low solubility and by the irreversible nature of the process.^7^ Alternatively, the characterization of the vertical ionization potential of single and small oligonucleotides has been addressed by means of the photoelectron spectroscopy (PES) technique.^10,11^ Although such an approach does not suffer from the limitations described above, for example, the vertical ionization energy (VIE) is measured from the instantaneous electron detachment, PES is not able to provide an estimate of the free energy of the process. For these reasons, the characterization of the DNA oxidation has stimulated the applications of several theoretical–computational approaches.^12−26^ In these studies, the quantum mechanical calculations on the nucleobases are performed in the gas phase and the effect of the environment is taken into account by means of dielectric continuum models. Despite the numerous models used to treat the effect of the environment, good estimates of the redox potentials of the nucleobases might require (i) the careful calibration of the method used to mimic the solvent effects^12,15^ and/or (ii) the explicit treatment of few solvent molecules.^16,20,21^ Here, we present a statistical–mechanical sound approach where the effect of the perturbation is treated by means of the perturbed matrix method,^27,28^ which combines the extended sampling as provided by classical molecular dynamics (MD) simulations and high-level quantum mechanical calculations. Similar to the other theoretical–computational approaches, we also divide the overall system into a subpart to be treated at the quantum mechanical level (the quantum center) and the remaining part (the environment), which is modeled as a purely classical atomistic system. However, compared to methods based on the use of continuum solvent models, our approach essentially differs in its ability to provide the atomistic behavior of the perturbation, hence reconstructing the dynamics of the perturbed quantum properties and thus the relevant effects of the fluctuations for both thermodynamics and kinetics. On the other hand, QM/MM methods although similar in the ability of providing a rigorous atomistic dynamical description of the system are typically severely limited in the phase space sampling and, hence, are often unable to reach proper sampling for evaluating both the thermodynamics and kinetics of the system.

By such an approach, it is therefore possible to estimate the dynamical effects of the environment molecules on the investigated quantum center (QC) and to calculate the free energy difference involved in a quantum state transition directly from the PMM calculations at a limited computational cost.

## Theory

### Perturbed Matrix Method

The MD-PMM approach is a hybrid quantum/classical theoretical–computational approach, similar in spirit to other hybrid methods,^29−33^ based on MD simulations and on the PMM. In this computational strategy,^27^ the part of the system in which the quantum processes of interest occur (the QC, i.e., in the present case, the redox center) is treated at the quantum level, and the effect of the rest of the system (the environment) on the properties of the QC is included as an electrostatic perturbation exploiting the atomistic configurations of the system sampled by classical MD simulations.

The electronic properties of the isolated QC (unperturbed properties) are calculated quantum chemically in vacuum (i.e., in the gas phase), and then, for each configuration generated by all-atom classical MD simulations of the whole system, the electrostatic effect of the instantaneous atomistic configurations of the environment is included as a perturbing term within the QC Hamiltonian operator. The electronic Hamiltonian operator *Ĥ* of the QC embedded in the perturbing environment can be thus expressed via
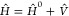
1where *Ĥ*^0^ is the QC unperturbed electronic Hamiltonian (i.e., as-obtained considering the isolated QC) and *V̂* is the perturbation operator. In typical PMM calculations, the perturbing electric field provided by the environmental atomic charges is used to obtain the perturbation operator, *V̂*, via a multipolar expansion centered in the QC center of mass, **r**_0_
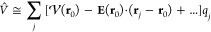
2with *j* running over all QC particles (i.e.,, nuclei and electrons); *q*_*j*_ is the charge of the *j*th particle, **r**_*j*_ is the corresponding coordinates,  is the electrostatic potential exerted by the perturbing environment, and **E** is the perturbing electric field . In the present work, a recent development of the PMM approach is used including higher order terms by expanding the perturbation operator around each atom of the QC (atom-based expansion).^28^ Within such an approach, the perturbation operator *V̂* is expanded within each *N*th atomic region around the corresponding atomic center **R**_*N*_ (i.e., the nucleus position of the *N*th atom of the QC), providing
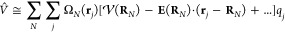
3with *j* running over all QC nuclei and electrons and *N* running over all QC atoms; Ω_*N*_ is a step function being null outside and unity inside the *N*th atomic region. The atom-based expansion is used here only for the Hamiltonian matrix diagonal elements, whereas the other Hamiltonian matrix elements are obtained using the QC-based perturbation operator expansion within the dipolar approximation (eq 2). At each frame of the MD simulation, the perturbed electronic Hamiltonian matrix is constructed and diagonalized, providing a continuous trajectory of perturbed eigenvalues and eigenvectors to be used for evaluating the QC instantaneous perturbed quantum observable of interest as, in the present case, the QC ground-state energy in the reduced and oxidized states.

### Redox Free Energy and Average Vertical Transition Energy

The Helmholtz free energy change Δ*A* providing the redox (reduction) free energy can be expressed as
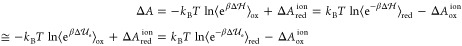
4in the abovementioned equation,  is the QC environment whole energy change upon oxidation (red → ox), with  being the corresponding QC perturbed electronic ground-state energy change (note that the electronic energy change is obtained at each classical configuration relaxing the quantum nuclear degrees of freedom and thus approximates the vibronic ground-state energy change, i.e.,  is the adiabatic ionization energy, AIE). The angle bracket subscripts ox and red indicate that both the energy change and the averaging are obtained either in the oxidized or reduced ensemble, respectively, each involving its own ionic condition, and the approximation  is used, that is, the environment internal energy change associated with the QC reaction is disregarded (being exactly zero when considering typical MD force fields and assuming the environment electronic state independent of the QC oxidation state).

Finally, Δ*A*_red_^ion^ is the relaxation free energy for the reduced species due to the ox → red ionic environment transition and Δ*A*_ox_^ion^ is the relaxation free energy for the oxidized species due to the red → ox ionic environment transition. From eq 4, it follows that  and  provide the upper and lower bounds of Δ*A* and hence, assuming Δ*A*_red_^ion^ ≈ Δ*A*_ox_^ion^, it can be written
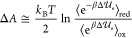
5

In this last equation, the perturbed electronic ground-state energy change and the ensemble averages are evaluated via the MD-PMM approach described in the previous subsection, with the two ensembles obtained by MD simulations involving the proper ionic environment, that is, each with the a proper number of counterions to neutralize the total charge of the MD simulation box.

Using the same notation and approximations, the average vertical transition energy in the reduced ensemble, that is, the VIE, can be expressed by , with the angle bracket subscript red indicating again that the energy change and the averaging are obtained within the reduced ensemble condition, although now, the electronic ground-state energy change  corresponds to the vertical electronic transition energy (i.e., the energy change is obtained at each classical configuration at fixed red quantum nuclear degrees of freedom, thus approximating the vertical vibronic energy change).

## Methods

Unperturbed energies and dipole moments for the ground state of the nitrogenous bases were calculated by means of density functional theory (DFT) using the B3LYP functional and the 6-311++G(2d,2p) basis set. The unperturbed electronic properties for the first six excited states were estimated, with the same functional and basis set, by means of time-dependent DFT. All the quantum mechanical calculations have been done by means of Dalton software. The optimized geometry used for the four nucleobases in their neutral and radical cation state was taken from the work of Psciuk et al.^12^ Note that two additional DFT functionals were tested for the unperturbed VIE calculations, in order to estimate the noise due to the DFT functionals. From these data (see the Supporting Information), we obtained a standard deviation of ≈0.3 eV. All the PMM calculations have been performed using the nitrogenous bases as QCs. MD simulations for the DNA deoxynucleosides (in both neutral and radical cation states), deoxynucleoside monophosphate, and dinucleotides were performed using the Gromacs software package^34^ and the AMBER99 force field.^35^ The MD simulations were performed in a cubic box of 3.1 nm sides at 300 K for 100 ns using a time step of 2 fs at a constant volume using the SPC model for the ≈1050 water molecules. Additional simulations of the DNA deoxynucleosides have been performed in acetonitrile^36^ for a further comparison with the available experimental data.^7^ The velocity-rescaling algorithm has been used to keep the temperature constant at 300 K.^37^ For the simulation of the deoxynucleoside radical cations, the atomic partial charges were estimated by the same procedure used for the estimation of the parameters in the AMBER force field.^35^

The reduction process considered is
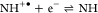
and the corresponding standard redox potential (i.e., the reduction potential *V*_red_) of the solvated molecule is defined as
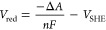
6where *F* is the Faraday constant and *n* is the number of electrons involved in the reaction. The value of the standard hydrogen electrode potential *V*_SHE_ was taken from the literature (4.281 V).

The estimates of the statistical error for the properties calculated in this work (i.e., VIE and *V*_red_) were performed by the block-averaging procedure. That is, the value of the observable interest was calculated in three subparts of the MD trajectory and the standard deviation of the mean was taken as a measure of the statistical inaccuracy of the observable.

## Results and Discussion

The optimized geometries of the nucleobases in both neutral and oxidized forms were calculated using the same procedure proposed in recent work.^12^ From these geometries, the unperturbed (gas-phase) properties, that is, excitation energies (see the Supporting Information) and dipole moments, have been calculated and used in the PMM to provide the perturbed properties, as explained in the Theory section.

### Vertical Ionization Energies

The VIEs for the nucleosides, nucleotides, and dinucleotides have been calculated by means of the PMM using the unperturbed electronic properties of the nitrogenous bases and the MD simulations of the molecules in the neutral state in water . As shown in Table 1, the effect of the environment, including the deoxyribose, is to lower the VIEs with respect to the gas-phase (unperturbed) values.^38−40^ The extent of such an effect depends on the nature of the nucleobases, varying between 0.26 eV for the guanosine and 0.68 eV for the thymidine.

**Table 1:** VIEs (in eV)^a^

molecule	VIE gas calcd	VIE gas exp^38^	VIE aq calcd	VIE aq corr.	VIE aq exp^11^
Nitrogenoues Bases					
guanine	7.92	8.24			
adenine	8.21	8.44			
thymine	8.97	9.14			
cytosine	8.71	8.94			
nucleosides					
guanosine			7.66	7.98	
adenosine			7.94	8.17	
thymidine			8.29	8.46	8.1
cytidine			8.26	8.49	8.1
Deoxynucleotides Monophosphate					
dGMP			7.57	7.89	7.3
dAMP			7.86	8.09	7.6
dTMP			8.22	8.39	
dCMP			8.17	8.40	7.9
Dinucleotides					
GG (5′)			7.59	7.91	
GG (3′)			7.56	7.88	
AA (5′)			7.85	8.08	
AA (5′)			7.79	8.02	
TT (5′)			8.27	8.44	
TT (3′)			8.28	8.45	
CC (5′)			8.23	8.46	
CC (3′)			8.20	8.43	

a The statistical error for the calculated values is 0.1 eV; the reported experimental uncertainty is 0.1 eV.^11^ Values calculated by means of PMM using ab initio energies (calcd) and using corrected unperturbed energies (corr.) are shown; see text.

The VIEs calculated for the (deoxy)nucleotide monophosphate are very similar to the corresponding nucleoside values, indicating that the presence of the phosphate group stabilizes the cation to a minor extent (∼0.1 eV). This result is in agreement with the PES measurements for the single case where this variation (0.2 eV) was provided, that is, cytidine. Both the VIEs of the nucleoside and nucleotide monophosphate present the same trend, with the guanosine having the lower VIE, followed by adenosine, cytidine, and thymidine (see Table 1).

Interestingly, the thymidine and deoxythymidine monophosphate (dTMP) both show a decrease in the VIE in water with respect to the gas phase significantly larger (0.68 and 0.75 eV, respectively) than the corresponding values for the other molecules. This effect is due to the variation in the dipole moment of the nucleobase; in fact, the thymine radical cation shows an increase in the unperturbed dipole moment of 1.8 debye with respect to the neutral form, whereas its increase is very limited (≃ 0.1 debye) in the adenine, cytosine, and guanine. Such an effect is then reflected on the perturbed energies of the radical cation, which remarkably decreases in the case of the thymidine and dTMP.

The VIE estimates for both nucleoside and deoxinucleotide monophosphate in aqueous solution are in rather good agreement with the corresponding experimental data evaluated by means of PES.^11^ In Table 1, we show the VIEs calculated by PMM either using the ab initio unperturbed energies (calcd) or correcting such energies by adding the shift between the unperturbed (gas-phase) experimental and calculated VIEs (corr.). Although we slightly overestimate aqueous VIEs with respect to the corresponding experimental values, the differences between these values are very close to the experimental estimates. In fact, our data estimate virtually identical VIE values for thymidine and cytidine in agreement with the experimental results. The same trend is observed in the case of the deoxinucleotide monophosphate, where the dGMP VIE is 0.3 eV lower than the dAMP VIE, which is in turn 0.3 eV lower than the dCMP VIE. Our corresponding estimates of the VIE differences are 0.29 and 0.41 eV (these differences remain quite unaffected when the experimental VIEs estimated in the gas phase were used; see Table 1).

To better understand how the presence of an additional nucleotide affects the VIE, we apply the same procedure to evaluate the VIEs for the di-homonucleotides AA, CC, GG, and TT. Within the accuracy of our calculations, the values of the VIEs, for both the 5′ and 3′ ends, are practically indistinguishable from the corresponding mononucleotide values, indicating that at least for dinucleotides, the VIEs depend on the chemical nature of the nucleobases only. This result is in line with a combined experimental–computational work,^41^ where the effect of the DNA surrounding was estimated to be negligible in an aqueous environment.

**Figure 1:**
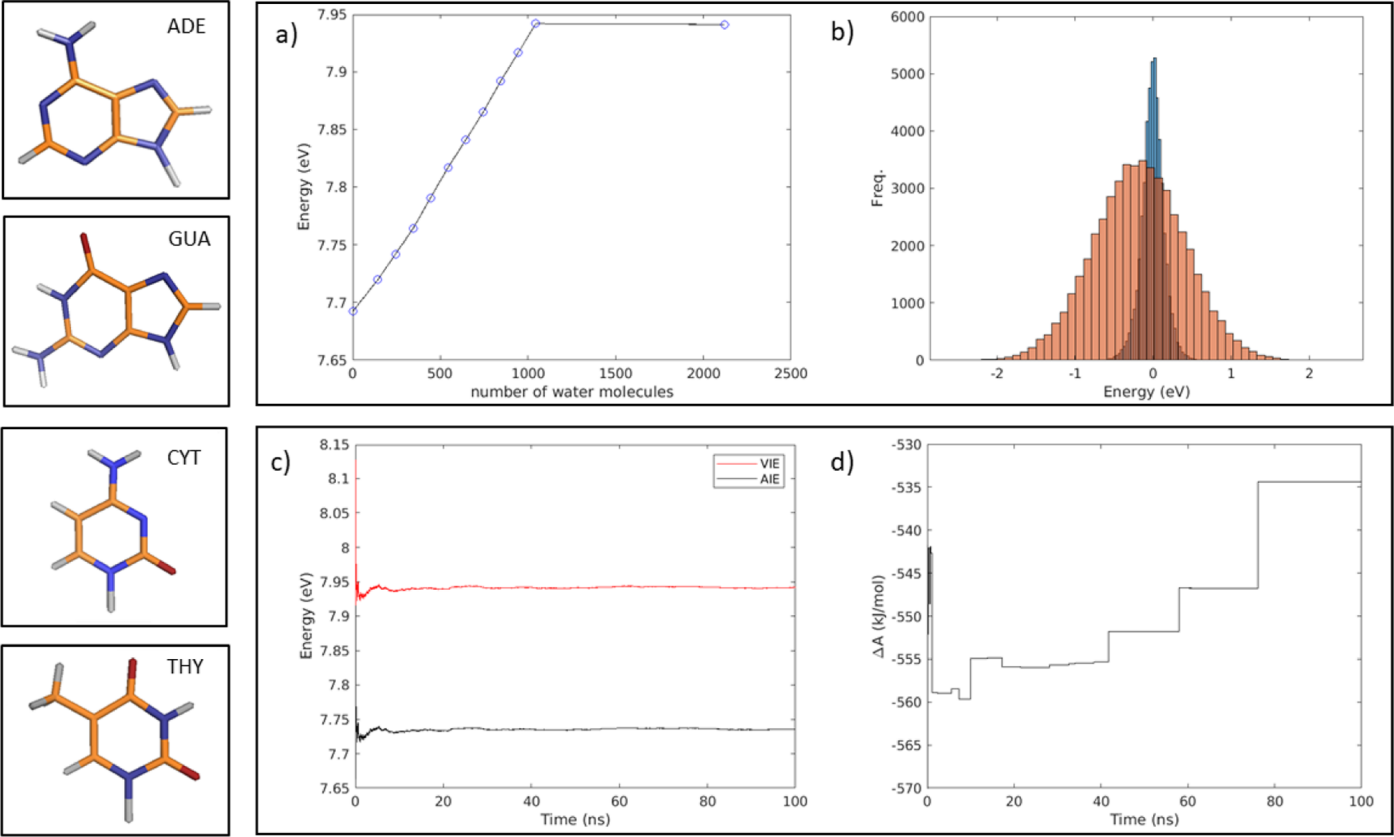
Convergence of the redox properties for adenosine. (a) Effect of the number of water molecules included in the calculation on the estimated VIE. (b) Distributions of the differences between the vertical oxidation energies as obtained in the reference simulation by removing 50 (orange) or 700 (blue) water molecules with respect to the VIE values including all (1044) the water molecules. (c) Estimated values of the AIE (black line) and VIE (red line) vs the MD trajectory length. (d) Estimated value of the reduction free energy Δ*A* vs the MD trajectory length.

The sensitivity of the VIE values to the water model was tested by performing an additional MD simulation of the dihomonucleotide GG using the SPC/E water model. Such a simulation provided VIE estimates indistinguishable, within the noise, from the values obtained using the SPC model. Furthermore, the convergence of the ionization energies with respect to the system size was checked by performing an additional MD simulation doubling the number of water molecules and the box volumes. These data, reported in Figure 1, show that the use of at least ∼1000 water molecules guarantees an appropriate estimate of such a property.

### Reduction Potentials

The estimate of the reduction potentials of the nucleobases required the calculation of the perturbed energies in both the neutral and cationic ensembles, as explained in the Theory section. For each ensemble using the nucleobases as a QC, the difference of the perturbed energies between the radical cation and the neutral species has been used in eq 5 to provide the reduction free energies. As expected, the effect of the environment, which includes the solvent and the counterions, is to stabilize the oxidized form (radical cation), such an effect being much more pronounced in the cationic ensemble (see Table 2). In this ensemble, the energies of the nucleosides are lowered in the range of 5.64 eV in the case of the adenosine in acetonitrile and to 6.57 eV in the case of the guanosine in water.

**Table 2:** Effects of the Environment on the Mean Electronic Energies for the Neutral  and Radical Cation States , with, , the Unperturbed (Gas Phase) Electronic Ground-State Energy

molecule	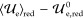 (eV)	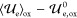 (eV)
Neutral Ensemble Water Solution		
guanosine	–2.62	–2.98
adenosine	–1.90	–2.18
thymidine	–1.91	–2.61
cytidine	–2.31	–2.75
Radical Cation Ensemble Water Solution		
guanosine	–1.18	–6.57
adenosine	–1.22	–5.98
thymidine	–1.39	–6.47
cytidine	–1.21	–6.32
Neutral Ensemble ACN Solution		
guanosine	–1.82	–2.37
adenosine	–1.33	–1.75
thymidine	–1.29	–2.06
cytidine	–1.38	–2.02
Radical Cation Ensemble ACN Solution		
guanosine	–1.92	–6.52
adenosine	–1.12	–5.64
thymidine	–1.31	–6.34
cytidine	–1.27	–6.23

As described in the Theory section, the calculation of the reduction potentials requires the knowledge of the reduction free energy, which in turn requires proper sampling of the process. Therefore, we first check that by means of our theoretical–computational approach, it is possible to achieve a good convergence of such a property. As reported in Figure 1, the free energy value reaches a reasonable convergence within the simulation time length, assuring that our sampling is wide enough for our purpose. Moreover, we also investigated the effect of considering the nitrogenous bases as QCs in the PMM, leaving the sugar ring as a perturbation. To this end, we compare the AIEs of the nitrogenous bases perturbed by the sugar ring as obtained by means of the PMM with those obtained by ab initio calculations of the whole nucleosides. These differences, all ≃0.1 eV, confirm that the choice of the nitrogenous bases as QCs provides the electronic properties with sufficient accuracy (data not shown).

The obtained reduction potentials of the deoxynucleosides are reported in Table 3 with the corresponding experimental estimates as provided by means of cyclic voltammetry.^7,42−44^ In order to reduce possible inaccuracies due to the ab initio calculations, the reduction potentials were obtained by PMM using the unperturbed energy correction, as described for the calculation of the VIEs, that is, correcting the unperturbed energies by adding the shift between the unperturbed (gas-phase) experimental and calculated AIEs, as provided (approximately) by the unperturbed VIE shift.

**Table 3:** Comparison between the Calculated (PMM) and Experimental (CV) Standard Reduction Potentials in Water and Acetonitrile (in V and vs SHE)^a^

	*V*_red_ (PMM)	*V*_red_ (CV)^42,45^	*V*_red_ (CV)^44^	*V*_red_ (CV)^43^
molecule	deoxynucleosides	nucleosides	deoxynucleotides	nucleobases
Water Sol.				
guanosine	1.05 (−0.21)	1.47 (−0.14)	1.49 (−0.10)	1.22 (−0.27)
adenosine	1.26 (0)	1.61 (0)	1.59 (0)	1.49 (0)
thymidine	1.73 (0.47)	1.90 (0.29)	1.65 (0.06)	1.49 (0)
cytidine	1.87 (0.61)	1.78 (0.17)	1.68 (0.09)	1.62 (0.13)

	*V*_red_ (PMM)	*V*_red_ (CV)^7^
molecule	deoxynucleosides	deoxynucleosides
Acetonitrile Sol.		
guanosine	1.18 (−0.32)	1.38 (−0.47)
adenosine	1.50 (0)	1.85 (0)
thymidine	1.64 (0.14)	2.00 (0.15)
cytidine	1.79 (0.29)	2.03 (0.18)

a The differences of the reduction potential of the molecule with respect to the adenosine is reported between parentheses.

Our results confirm the experimental trend, the guanosine being the nucleoside with the lowest *V*_red_ in water, followed by adenosine, thymidine, and cytidine. In particular, our theoretical–computational procedure provides the same behavior with respect to the cyclic voltammetry measurements, the guanosine being the most ionizable among the four nucleosides and the thymidine and cytidine being the nucleosides with the highest reduction potentials. The differences between thymidine and cytidine are very small and, within the experimental–computational error, their reduction potentials are almost indistinguishable as provided by our computational procedure and by cyclic voltammetry. In fact, we estimated an experimental error of 0.2 V as calculated by the standard deviation between the different values reported in the literature, whereas our statistical error corresponds to 0.1 V as provided by the block-averaging procedure. Therefore, our estimates of the reduction potentials in water reasonably well reproduce the experimental values,^42−44^ with deviations always lower than 0.5 V for all the molecules. Similar results are obtained in acetonitrile solution, confirming the good reproduction of the experimental data^7^ (see Table 3). Interestingly, our results obtained without any semi-empirical fit are similar to the theoretical–computational values as obtained by means of the widely used polarizable continuum model, where the solvent cavity parameters were *adjusted to improve the agreement with the experimental results*.^12^ A detailed comparison between our results and the available computational data (showing the extent of the variation in the calculated standard reduction potentials) is reported in the Supporting Information.

## Conclusions

MD simulations and quantum mechanical calculations have been combined through a robust theoretical approach to estimate the VIEs and the reduction potentials of nucleic acids in solutions. The values obtained for the VIEs, reasonably close to the experimental ones, showed the expected stabilization of the radical cation forms. Such an effect is more pronounced in the case of thymidine and dTMP, where the dipole moment of the thymine radical cation significantly increases with respect to the neutral form. Noteworthy, the effect of the environment is mainly due to the solvent, the contribution of the additional nucleotide in dinucleotides and of the phosphate group being almost negligible. The application of the same theoretical–computational procedure also allowed us to evaluate the reduction potentials of the nucleosides thymidine, cytidine, adenosine, and guanosine. Our data are in line with the most recent electrochemical estimates of the reduction potentials both in water and acetonitrile solutions. In particular, guanosine, the most easily oxidized site along DNA strands, is the nucleoside with the lowest reduction potential, in agreement with previous experimental data. Future studies will address the effect of the environment in a single- or double-stranded DNA context to highlight the role of the sequence in the ionization process.

## Supporting Information Available

Excitation energies of the nucleobases, comparison of the ionization energies for different DFT functionals, and comparison with available computational estimates of the standard reduction potentials (PDF)
