# Supplementary material for: Theoretical Characterization of the Reduction Potentials of Nucleic Acids in Solution
Source: J Chem Theory Comput. 2021 Feb 23;17(3):1301–7. doi: 10.1021/acs.jctc.0c00728 (PMC8028051; doi:10.1021/acs.jctc.0c00728)
Supplement: Supplementary file 1 — ct0c00728_si_001.pdf [file ct0c00728_si_001.pdf]

Theoretical Characterization of the reduction Potentials of nucleic acids in solution

Valeria D'Annibale,[†] Alessandro Nicola Nardi,[†] Andrea Amadei,[*,‡] and Marco D'Abramo[*,†]

†*Department of Chemistry, Sapienza University of Rome, Rome, Italy*

‡*Department of Chemical Sciences and Technology, Tor Vergata University, Rome, Italy*

E-mail: andrea.amadei@uniroma2.it; marco.dabramo@uniroma1.it

Supporting Information

1.1 Comparison of Vertical Ionization Energies (VIE) as obtained by different DFT functionals

VIEs of nucleobases have been obtained using different functionals: B3LYP, M06-2X and BP86.

Base	VIE (eV) B3LYP / 6-311++G(2d,2p)	VIE (eV) M06-2X / 6-311++G(2d,2p)	VIE (eV) BP86 / 6-311++G(2d,2p)
Adenine	8.21	8.48	8.85
Guanine	7.92	7.53	7.96
Cytosine	8.71	8.96	9.29
Thymine	8.97	9.23	9.58

1.2 Excitation Energies

The Excitation Energies have been evaluated using TDDFT (B3LYP/6-311++G(2d,2p)).

1.2.1 Excitation Energies for neutral nucleobases

Exc State	Adenine (eV)	Guanine (eV)	Cytosine (eV)	Thymine (eV)
1	4.92	4.54	4.58	4.69
2	4.94	4.81	4.74	4.91
3	5.15	4.98	5.09	5.40
4	5.20	5.13	5.17	5.76
5	5.41	5.24	5.39	5.91
6	5.55	5.52	5.55	6.09

1.2.2 Excitation Energies for radical-cation nucleobases in the equilibrium geometry of neutral nucleobases

Exc. State	Adenine (eV)	Guanine (eV)	Cytosine (eV)	Thymine (eV)
1	0.79	1.31	0.45	0.59
2	1.26	1.59	0.59	1.14
3	1.67	1.99	1.00	1.36
4	2.07	2.12	3.07	3.25
5	2.58	2.90	3.85	3.68
6	3.69	2.99	4.75	4.08

1.2.3 Excitation Energies for radical-cations nucleobases in the equilibrium geometry of the relaxed radical-cation nucleobase

Ex. en. State n.	Adenine (eV)	Guanine (eV)	Cytosine (eV)	Thymine (eV)
1	1.06	1.69	0.56	1.11
2	1.83	2.08	0.75	1.79
3	2.10	2.58	1.19	1.99
4	2.54	2.73	3.08	3.79
5	2.96	3.39	4.24	4.11
6	4.00	3.41	5.00	4.37

1.3 Standard reduction potentials of deoxynucleosides in water as obtained by PMM procedure and by different theoretical-computational methods reported in literature

Base	V_{red} (V) PMM *Deoxy nucleoside*	V_{red} (V) Psciuk et al.[1] *Nucleoside*	V_{red} (V) Li et al. *Nitrogenous base*	V_{red} (V) Paukku, Hill.[2] *Nitrogenous base*	V_{red} (V) Wang et al.[3] *Nitrogenous base*	V_{red} (V) Wang et al.[3] *Deoxy nucleoside*
**Guanine**	1.05	1.21	1.44	1.49	2.03	2.08
**Adenine**	1.26	1.52	1.71	1.86	2.38	2.44
**Thymine**	1.73	1.81	1.78	2.09	2.57	2.78
**Cytosine**	1.87	1.95	2.10	2.19	2.68	2.77

1.4 Standard reduction potentials of deoxynucleosides in acetonitrile solution as obtained by PMM procedure and by different theoretical-computational methods reported in literature

Base	V_{red} (V) PMM *Deoxy nucleoside*	V_{red} (V) Crespo-Hernandez et al.[4] *Nitrogenous base*	V_{red} (V) Crespo-Hernandez et al.[4] *syn-Deoxynucleoside*	V_{red} (V) Crespo-Hernandez et al. [4] *anti-Deoxyucleoside*
**Guanine**	1.18	2.04	2.04	1.82
**Adenine**	1.50	2.20	2.17	1.99
**Thymine**	1.64	2.62	2.41	2.24
**Cytosine**	1.79	2.44	2.34	2.12

The standard redox potentials are all reported against the reference standard hydrogen electrode ($V_{SHE} = 4.281\ V$).

BIBLIOGRAPHY

1. Psciuk, B. T., Lord, R. L., Munk, B. H. & Schlegel, H. B. Theoretical determination of one-electron oxidation potentials for nucleic acid bases. in *Journal of Chemical Theory and Computation* vol. 8 5107–5123 (2012).

2. Paukku, Y. & Hill, G. Theoretical determination of one-electron redox potentials for DNA bases, base Pairs, and stacks. *J. Phys. Chem. A* **115**, 4804–4810 (2011).

3. Wang, J., Yang, S. & Zhang, Y. One-electron oxidation and redox potential of nucleobases and deoxyribonucleosides computed by QM/MM simulations. *Chem. Phys. Lett.* **739**, 136948 (2020).

4. Crespo-Hernández, C. E., Close, D. M., Gorb, L. & Leszczynski, J. Determination of Redox Potentials for the Watson−Crick Base Pairs, DNA Nucleosides, and Relevant Nucleoside Analogues. *J. Phys. Chem. B* **111**, 5386–5395 (2007).
